# Engineering human peritoneum in vitro: A novel microfluidic platform for modeling peritoneal physiology and pathophysiology

**DOI:** 10.1002/btm2.70128

**Published:** 2026-03-09

**Authors:** Katharina Peisert, Franziska Keßler, Sara Y. Brucker, Jan Pauluschke‐Fröhlich, Peter Jakubowski, Felix Neis, Bernhard Krämer, Jürgen Andress, Adrian Weghofer, Hui‐Yu Liu, Peter Loskill, Martin Weiss

**Affiliations:** ^1^ Department of Women's Health Tübingen Eberhard Karls University Tübingen Tübingen Germany; ^2^ Department for Microphysiological Systems Institute of Biomedical Engineering, Faculty of Medicine Eberhard Karls University Tübingen Tübingen Germany; ^3^ NMI Natural and Medical Sciences Institute University Tübingen Reutlingen Germany

**Keywords:** 3D cell culture, fibrin gel, hydrogel, microfluidics, organ‐on‐chip, peritoneal adhesions, peritoneum

## Abstract

The peritoneum, the body's largest serous membrane, plays critical roles in abdominal homeostasis and immune defense. When disrupted by surgery or disease, it can lead to devastating complications including peritoneal adhesions—affecting up to 93% of surgical patients—peritonitis, and metastatic spread. Current research models fail to capture the complexity of human peritoneal biology, relying on inadequate animal models or oversimplified 2D cultures. Here, we introduce a PDMS‐free microfluidic platform that recreates the structural and functional architecture of human peritoneum. Our system combines immortalized mesothelial cells (MeT5A) with patient‐derived peritoneal fibroblasts in a physiologically relevant 3D environment, enabling real‐time analysis of peritoneal function and dysfunction. Through systematic evaluation of stromal matrices, we identified fibrin gel as optimal for supporting healthy mesothelial monolayer formation while maintaining excellent cell viability over 14 days. Importantly, we demonstrate the platform's translational potential by successfully modeling peritoneal adhesion formation. This innovative tool may improve the understanding of peritoneal biology, accelerating drug discovery and developing personalized treatment strategies for peritoneal diseases.

## Graphical abstract

Development of a first microfluidic peritoneum‐on‐chip platform that mimics the human peritoneal microenvironment enabling translational testing of therapeutic agents such as anti‐adhesion treatments under physiologically relevant conditions.

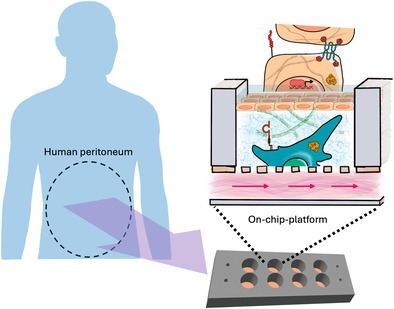

**Translational Impact Statement**

The lack of physiologically human peritoneal models has created a critical bottleneck in developing treatments for peritoneal diseases, including surgical adhesions that affect nearly all abdominal surgery patients. We present an innovative PDMS‐free microfluidic platform that recreates native peritoneal tissue architecture through systematic optimization of cellular and matrix components. This system successfully models pathological conditions, including adhesion formation, while enabling quantitative therapeutic testing under controlled conditions. The platform bridges the translational gap between laboratory research and clinical application, offering unprecedented opportunities for mechanistic studies, drug screening, and personalized medicine approaches.

## 1 INTRODUCTION

The peritoneum is the largest serous membrane in the human body which lines the abdominal and pelvic cavities, in addition to internal organs, to promote proper peristaltic movements, fertility and immune function.^1^, ^2^ Comprising both visceral and parietal components, this membrane system creates a protective environment for abdominal organs while facilitating their coordinated function.^2^, ^3^ Despite the differences in localization, the peritoneum shows a comparatively uniform structure.^2^

**FIGURE 1:**
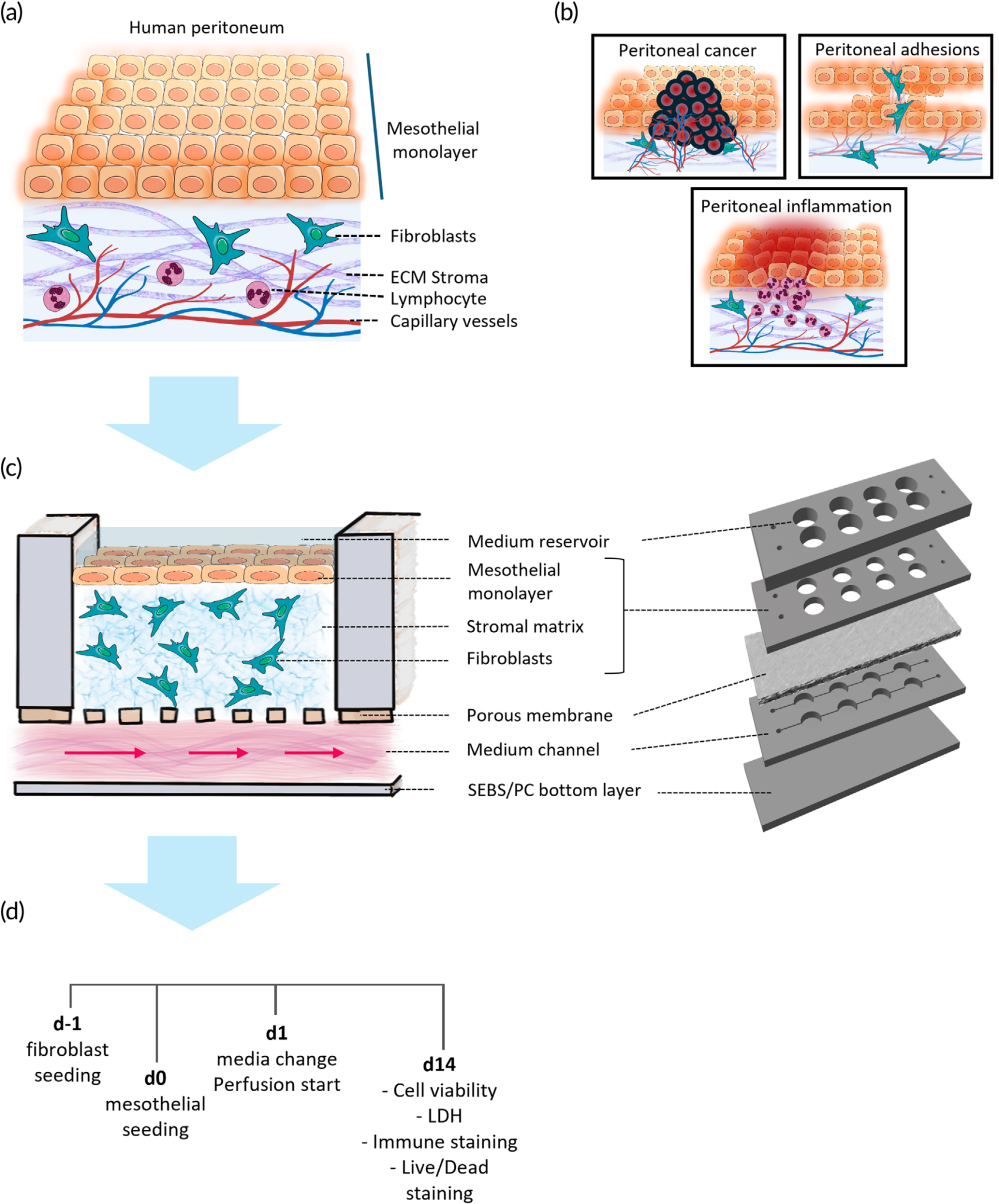
Schematic structure of the microfluidic platform of the human peritoneum. (a) Schematic representation of human peritoneal structure showing mesothelial monolayer overlying stromal components containing fibroblasts, ECM, immune cells, and vasculature. (b) Schematic representation of pathological conditions including peritoneal cancer, adhesions, and inflammation. (c) Cross‐sectional view (left) of the microfluidic platform design showing medium reservoir, mesothelial monolayer, stromal matrix with embedded fibroblasts, porous membrane, medium channel, and SEBS/PC bottom layer. Platform layering (right). (d) Cell seeding protocol and experimental timeline from day 1 (fibroblast seeding) through day 14 (analysis endpoints).

Its three‐layer architecture—mesothelial monolayer, basal lamina, and submesothelial stroma (Figure 1a)—enables a remarkable range of functions from friction reduction to immune defense. Under physiological conditions, the peritoneal cavity maintains only 5–20 mL of peritoneal fluid, yet processes approximately 1 L daily through its exceptional absorptive capacity.^2^, ^4^ The peritoneum plays a critical role in reducing friction, enabling passive and active transport of fluids and solutes and acting as a protective barrier, crucial for the intraabdominal homeostasis and tissue repair.^2^, ^3^, ^5^ This dynamic system depends on the delicate balance between mesothelial cells, which exhibit unique epithelial‐mesenchymal characteristics due to their mesodermal origin, and the underlying stromal network containing fibroblasts, immune cells, and vasculature.^6^, ^7^

When surgical intervention disrupts this delicate system, the consequences can be profound. Peritoneal adhesions—essentially internal scar tissue forming unwanted connections between organs—develop in 67%–93% of patients undergoing abdominal and gynecological surgery.^1^, ^8^ These adhesions transform successful operations into lifelong complications: chronic pain, secondary infertility, and life‐threatening bowel obstructions.^9^ Despite decades of research into pharmaceutical and barrier‐based prevention strategies, adhesion rates remain stubbornly high.^10^ Peritoneal carcinomatosis represents a terminal diagnosis for many cancer patients, while conditions like endometriosis exploit peritoneal biology to establish ectopic implants. Understanding these pathological processes requires models that capture the full complexity of human peritoneal physiology (Figure 1b).

To date the most described research approaches to mimic human peritoneum in vitro are animal‐ or 2D‐based studies.^10^, ^11^ Animal models, while valuable, exhibit significant physiological differences that limit their predictive value for human outcomes.^12^ Traditional 2D cell cultures, though useful for basic studies, cannot replicate the intricate cell‐matrix interactions and three‐dimensional architecture essential for peritoneal function.^13^ Currently, clinical research lacks tools for personalized treatment selection, and patients continue to suffer from preventable complications.

Here, we present a PDMS‐free microfluidic platform (Figure 1c,d) combining human peritoneal fibroblasts with mesothelial cells, mimicking the structural composition of the human peritoneum and enabling unprecedented investigation of peritoneal biology.

## 2 RESULTS

### 2.1 Cellular characterization reveals optimal marker profiles

**FIGURE 2:**
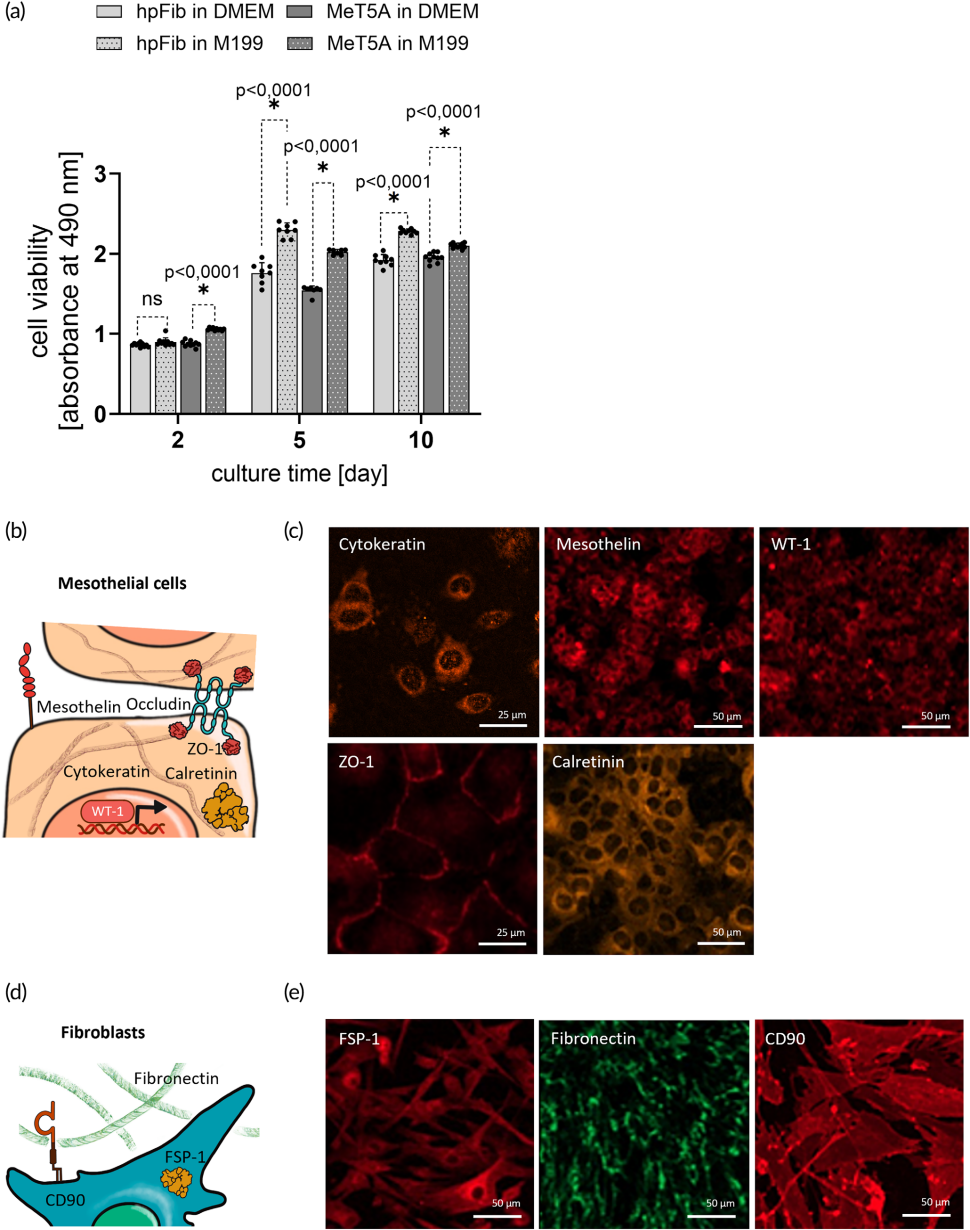
Antibody characterization of MeT5A and hpFib using immunofluorescence staining. (a) Cell viability of MeT5A and hpFib cultured in supplemented M199 or DMEM. Cell viability was assessed after 2, 5, and 10 days using an MTS assay. Absolute viability values are shown. For both MeT5A and hpFib cells, viability was significantly higher in supplemented M199 compared to supplemented DMEM at individual time points. Data are presented as mean ± SD, and individual values for each replicate are indicated (*n* = 8–10). *p < 0.05 (as determined by *t* test). (b) Schematic representation illustrates subcellular localization of mesothelial markers in MeT5A cells. (c) Representative immunofluorescence images show marker expression patterns after 24 h culture and 7 days for ZO‐1 tight junction maturation. Cytokeratin exhibits strong cytoplasmic/membrane localization. Anti‐mesothelin staining demonstrates a uniform cell surface expression pattern but exhibits low cell‐type specificity. Anti‐Calretinin staining showed a strong signal but a weak correlation to the nuclear staining. WT1 displays robust nuclear localization with high mesothelial specificity. ZO‐1 localizes to intercellular junctions, confirming barrier function. (d) Schematic representation illustrates subcellular localization of markers in fibroblasts. hpFib were cultured for 24 h. (e) Fibronectin shows strong but non‐specific expression in hpFib. CD90 demonstrates specific membrane localization in hpFib. FSP‐1 exhibits cytoplasmic but non‐specific distribution in hpFib. Scale bar: 25 and 50 μm.

Before platform development, we systematically characterized our cellular components to establish reliable identification markers (Figure 2). To assess medium compatibility for co‐culture applications, we evaluated the viability of MeT5A and hpFib cells when cultured in non‐optimal media conditions. Cell viability was quantified using MTS assay at 2, 5, and 10 days post‐seeding. Absolute cell viability values were shown for both cell types under each condition. For MeT5A and hpFib cells, viability was significantly higher in M199 than in DMEM (Figure 2a). Following this analysis, DMEM was used in all subsequent experiments for consistency. We systematically characterized both cell types using comprehensive immunofluorescence panels to identify cell‐specific markers. For MeT5A cells, we evaluated Cytokeratin (cytoskeletal component), Mesothelin (cell surface glycoprotein), Calretinin (calcium‐binding protein), WT1 (transcription factor), and ZO‐1 (tight junction protein, after 7‐day culture in M199 for junction maturation), while hpFib cells were assessed for Fibronectin (ECM component), CD90 (membrane glycoprotein), and FSP‐1 (calcium‐binding protein) expression (Figure 2b–e).

WT1 emerged as the most specific mesothelial marker, demonstrating strong nuclear localization in MeT5A with negligible background in hpFib. Also, Calretinin staining in MeT5A showed a strong cell‐type specific signal that was absent in hpFib (Figures 2c and S1, Supporting Information). Conversely, CD90 proved highly specific for fibroblast identification, showing robust membrane expression in hpFib while remaining undetectable in MeT5A (Figures 2e and S2). ZO‐1 staining confirmed functional tight junction formation in mature MeT5A cultures.

However, several traditionally used markers showed unexpected cross‐reactivity: Mesothelin, Fibronectin, and FSP‐1 were expressed in both cell types, though with varying intensities. These observations were confirmed by quantitative analysis of fluorescence signal intensities (see Figure S3), which showed trends consistent with the representative microscopy images. ZO‐1 staining was assessed qualitatively only and was not included in the quantitative analysis since fibroblasts lack ZO‐1 expression, precluding a meaningful quantitative comparison.

### 2.2 Fibrin gel emerges as superior matrix for peritoneal microfluidic modeling

**FIGURE 3:**
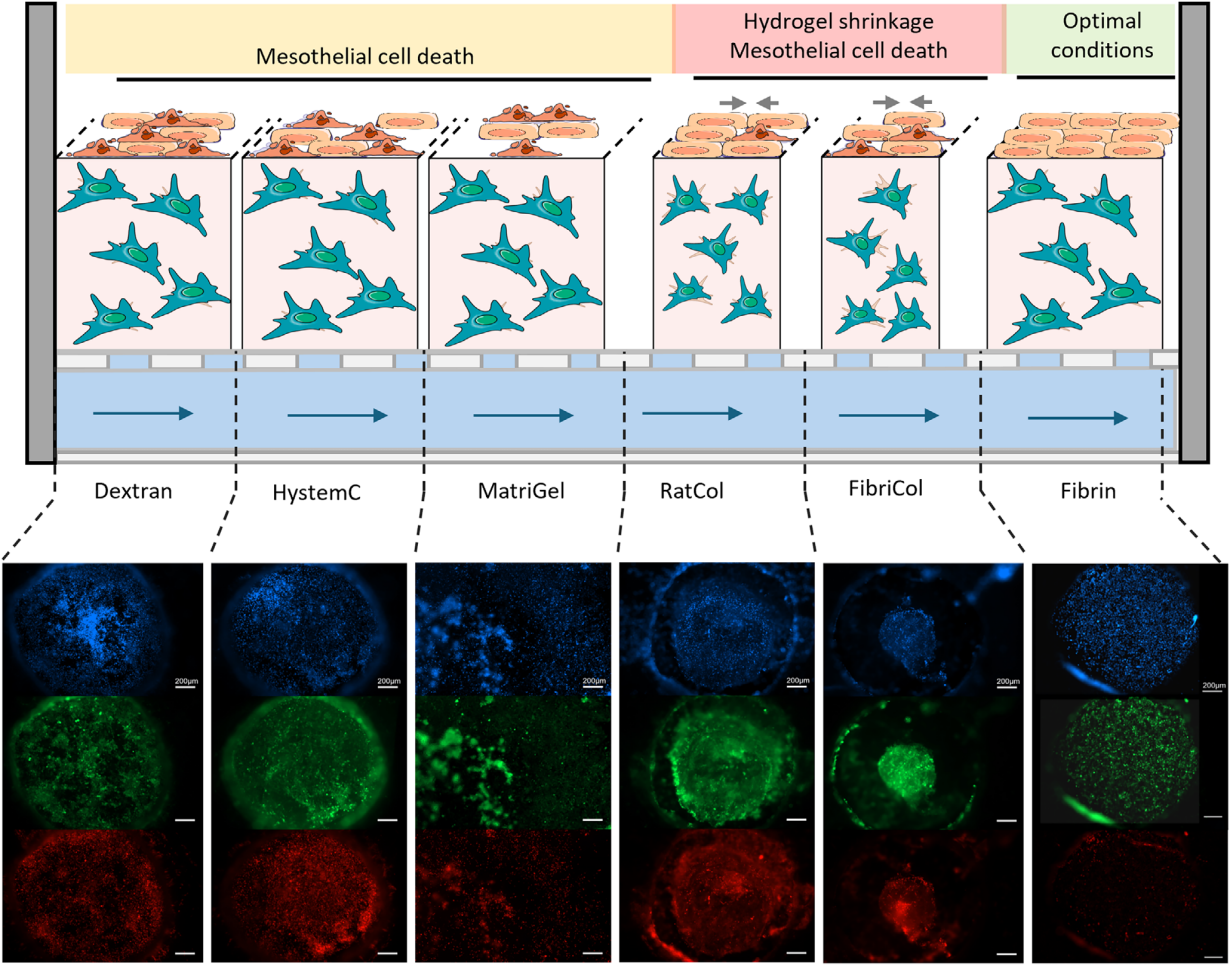
Matrix Optimization Reveals Fibrin Gel Superiority. Schematic representation and representative Live/Dead‐Stainings of MeT5A cell distribution and viability with different hydrogels after 5 days of cell culture. Cells were stained with Hoechst 33342 (blue) for nuclei, Calcein‐AM (green) for live cells, and propidium iodide (PI, red) for dead cells. Fibrin hydrogels achieved optimal mesothelial monolayer uniformity coupled with minimal cell death signals (low PI intensity). *n* = 3, scale bar: 200 μm.

To optimize stromal matrix composition, we systematically evaluated six hydrogel systems for their capacity to support physiologically relevant mesothelial monolayer formation and maintain cell viability. MeT5A cells were seeded atop hpFib‐embedded hydrogels covered with DMEM, and viability was assessed using Live/Dead staining (Calcein/PI) at 2, 5, and 8 days post‐seeding. Representative images at day 5 demonstrate distinct matrix‐dependent cellular responses (Figure 3). Dextran hydrogels exhibited high inter‐experimental variability with heterogeneous cell distribution patterns, characterized by alternating regions of confluent and sparse cell coverage. HyStem‐C supported relatively uniform cell spreading but demonstrated elevated PI fluorescence, indicating compromised cell viability. Matrigel promoted aberrant cell clustering rather than monolayer formation, with concentrated PI signal within aggregated regions suggesting localized cell death. Both collagen‐based matrices (RatCol, FibriCol) underwent significant dimensional shrinkage, causing detachment from platform periphery and reduced functional surface area. FibriCol contraction was more pronounced than RatCol, though FibriCol better supported uniform cell distribution. RatCol showed improved viability kinetics over time despite initial distribution heterogeneity. Fibrin gel emerged as the superior matrix, demonstrating optimal performance across all parameters: minimal PI signal (indicating high viability), uniform mesothelial monolayer formation, and dimensional stability without edge detachment. Table 1 summarizes these findings.

**TABLE 1:** Summary of results of utilized hydrogels.

Hydrogel	Result
Dextran Hydrogel	High variability between experiments, uneven cell distribution
HyStemC	Even cell distribution, low cell viability
MatriGel	Uneven cell distribution, clustering pattern, low cell viability
RatCol	Uneven cell distribution, shrinkage
FibriCol	Even cell distribution, shrinkage
Fibrin gel	Even cell distribution, high cell viability

### 2.3 Microfluidic media perfusion enhances platform performance and culture conditions

**FIGURE 4:**
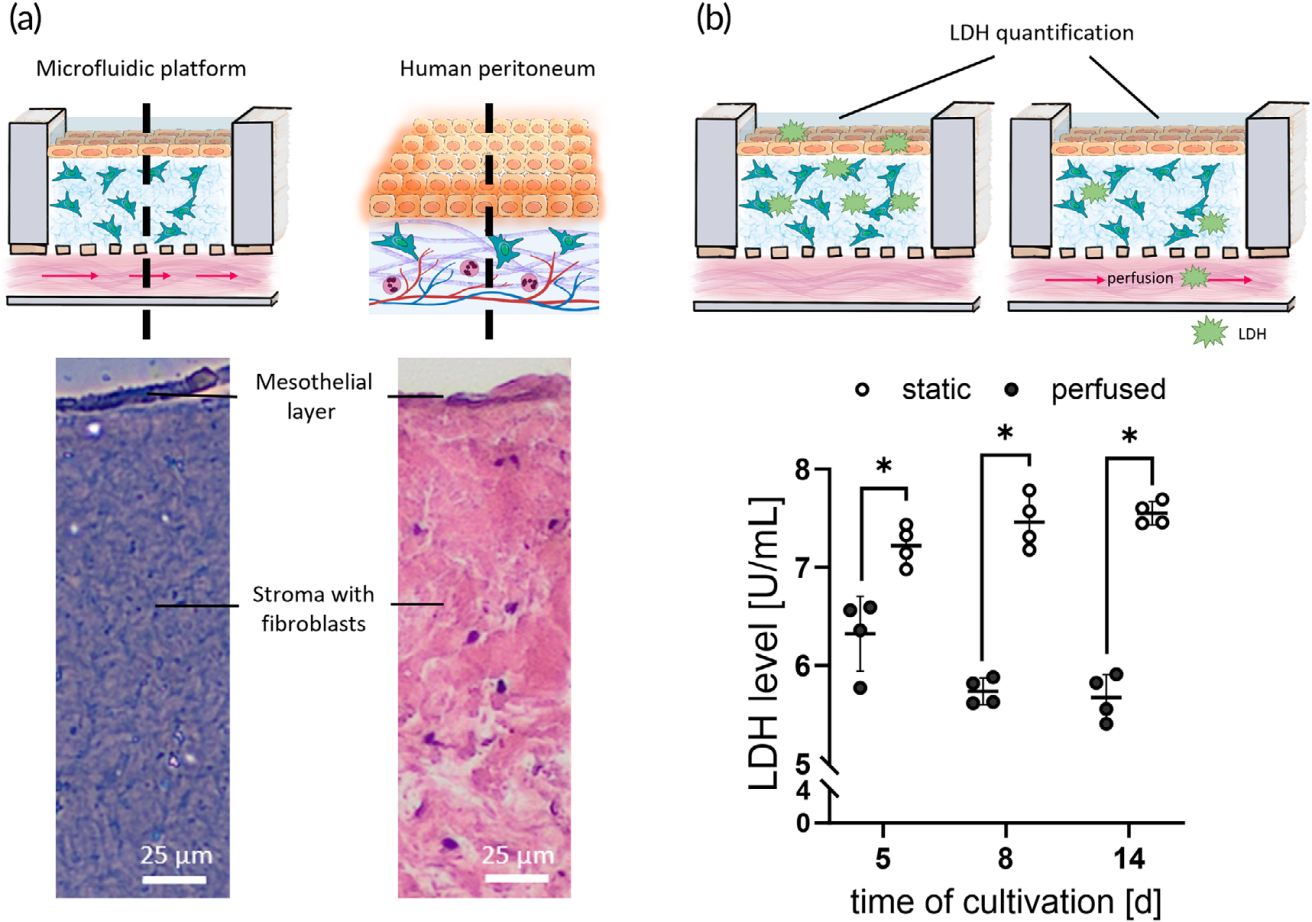
Functional and structural validation of the microfluidic peritoneum platform. (a) Representative H&E staining of microfluidic platform tissue (left) and native human peritoneum (right). Both samples demonstrate similar layered architecture, comparable cellular organization, layer thickness ratios, and cellular density gradients to native human peritoneum. Images acquired at 40× magnification. Scale bars: 25 μm. Representative images from *n* = 3 independent platform preparations and *n* = 2 human peritoneal samples. (b) LDH levels in effluent medium comparing static and perfused cultivation were measured after 5, 8, and 14 days of culture. Quantitative LDH levels are significantly higher in static cultivation compared to perfused cultivation. Data are shown as the mean ± SD, and individual values for each replicate are indicated (*n* = 4), *p < 0.05 (as determined by unpaired *t* test).

Histological comparison between our platform and native human peritoneum using H&E staining revealed similar architectural features (Figure 4a). Both the microfluidic platform and human peritoneum displayed a dense, hematoxylin‐rich surface layer corresponding to the mesothelial monolayer and an underlying eosin‐rich stromal region containing extracellular matrix and fibroblast populations. Clear morphological delineation between the epithelial and stromal compartments was observed in both samples. The platform tissue demonstrated comparable cellular organization and matrix distribution patterns to native human peritoneum, with similar layer thickness ratios and cellular density gradients.

To assess the impact of dynamic culture conditions on tissue viability, we performed LDH analysis after 5, 8, and 14 days of culture comparing static versus perfused platforms. Lactate dehydrogenase, a cytoplasmic enzyme released upon plasma membrane compromise, was measured as a biomarker for cellular damage and tissue integrity. Static cultures showed progressively increasing LDH release over time, with levels rising significantly by day 14 (*p* = 0.0028, Figure 4b). This progressive increase indicated accumulating cellular stress and membrane destabilization in non‐perfused conditions. Perfused platforms demonstrated markedly different kinetics, with LDH levels decreasing over the culture period. This declining pattern indicated enhanced membrane integrity and reduced cellular stress compared to static conditions. The temporal LDH profiles revealed superior cell survival and tissue stability under perfused conditions, with minimal membrane damage throughout the 14‐day culture period.

### 2.4 Bead‐based adhesion assay enables modeling and quantitative assessment of adhesion formation

Following successful recapitulation of healthy peritoneal architecture, we next evaluated the platform's capacity to model pathological conditions, focusing on peritoneal adhesions as a clinically relevant example. Peritoneal adhesions develop as maladaptive healing responses to surgical trauma, forming pathological fibrous connections between normally non‐adherent anatomical structures, including peritoneal membranes and visceral organs.

**FIGURE 5:**
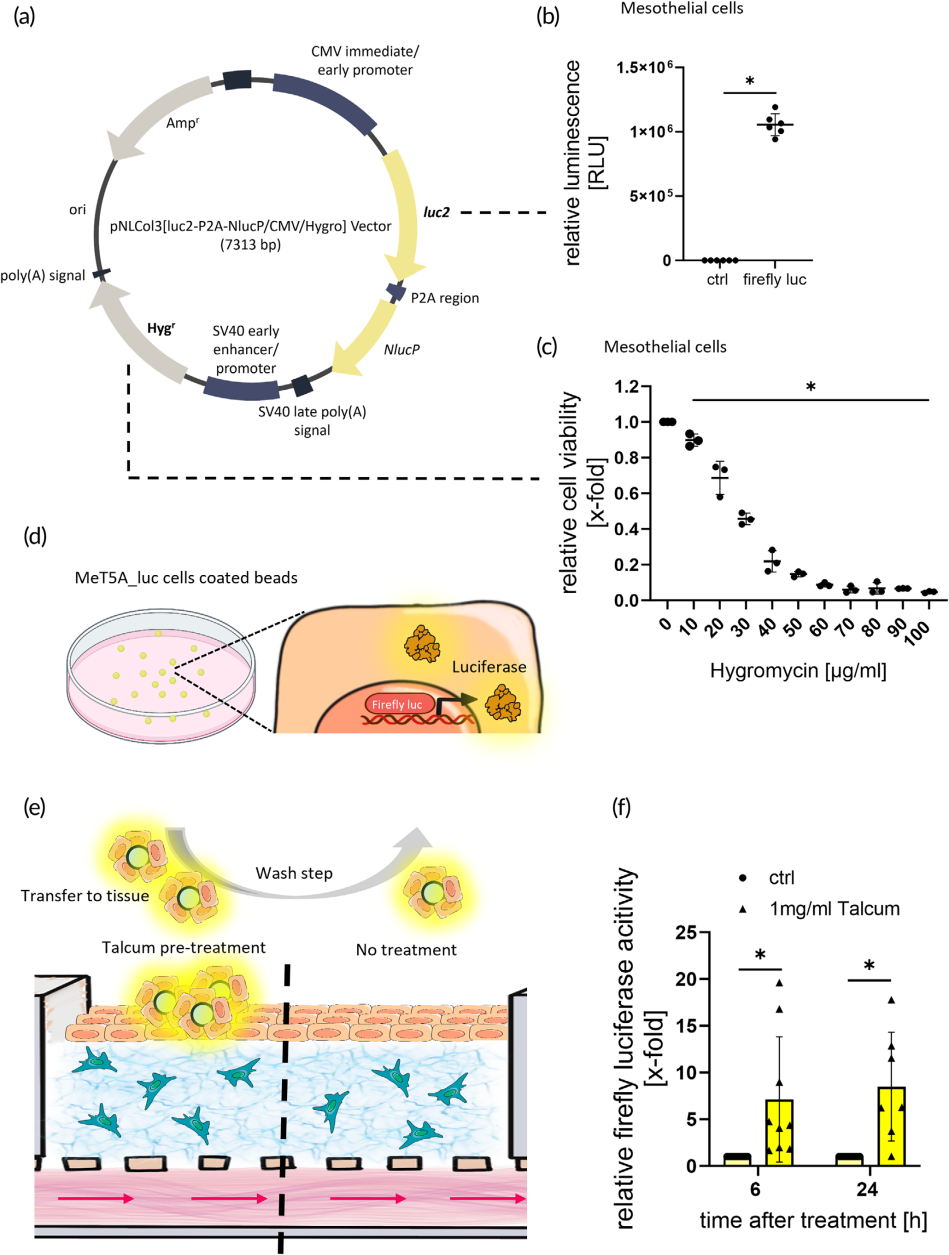
Bead‐based adhesion modeling enables quantitative assessment of adhesion formation. (a) Stable luciferase‐expressing cell line generation. The plasmid design includes luciferase gene and hygromycin resistance. (b) Successful transfection with significantly elevated luciferase activity in MeT5A_luc cells compared to control cells. (c) Hygromycin kill curve establishing 70 μg/mL as optimal selection concentration. (d) Schematic representation of bead coating process showing MeT5A_luc cells expressing firefly luciferase coating dextran beads. (e) Schematic representation of adhesion assay workflow showing bead transfer to tissue platform, talcum treatment (adhesion inducer) versus no treatment control, washing step to remove non‐adherent beads, and luciferase quantification. (f) Quantitative assessment of adhesion formation showing significant increases in relative firefly luciferase activity at both 6 h (7‐fold increase) and 24 h (9‐fold increase) following talcum treatment compared to untreated controls (*p < 0.05). Data are shown as the mean ± SD, and individual values for each replicate are indicated, (b) *n* = 6, (c) *n* = 3, (f) *n* = 9, *p < 0.05 (as determined by unpaired *t* test for (b) and one‐way ANOVA for (c) and (f)).

First, we successfully generated stable MeT5A_luc cells with robust luciferase expression, enabling quantitative tracking of cell attachment and adhesion formation using a dual‐reporter plasmid system (Figure 5a). Successful transfection was confirmed by significantly elevated luciferase activity in MeT5A_luc cells compared to parental controls (Figure 5b). Optimal hygromycin selection conditions were determined through dose–response analysis, identifying 70 μg/mL as the minimal concentration achieving complete selection after 72 h (Figure 5c). For adhesion modeling, dextran beads were coated with MeT5A_luc cells and cultured for 72 h to achieve confluent coverage (Figure 5d). These reporter beads were then transferred to established peritoneal platforms and treated with talcum powder, which is known to induce clinically relevant adhesions by triggering a local inflammatory response in mesothelial cells. The assay principle relies on differential bead retention: non‐adherent beads are removed during washing steps, while adherent beads remain attached to the tissue surface, enabling quantitative detection through luciferase activity (Figure 5e).

Talcum treatment resulted in significant bead adhesion, demonstrated by 7‐fold increased luciferase activity at 6 h, further rising to 9‐fold at 24 h compared to untreated controls (Figure 5f). This time‐dependent adhesion response nicely demonstrates the platform's capacity for modeling peritoneal adhesion formation and provides a system for in vitro evaluating anti‐adhesion therapeutic strategies in future.

## 3 DISCUSSION

We developed a PDMS‐free microfluidic platform that effectively recapitulates key aspects of human peritoneal architecture and function. This platform addresses an important need in biomedical research, as physiologically relevant in vitro models of the human peritoneum are limited, which has hindered efforts to understand and treat peritoneal diseases. The platform's PDMS‐free design eliminates well‐documented issues with small molecule absorption and leaching that plague conventional microfluidic platforms.^14^ This advancement proves particularly important for drug testing applications, where accurate pharmacokinetics are essential for meaningful results. Our identification of fibrin gel as the optimal stromal matrix aligns well with its established biological role in peritoneal physiology. Fibrin participates in natural peritoneal repair processes, providing a relevant substrate for modeling both healthy and pathological conditions.^15^ This matrix offers suitable mechanical properties, integrin binding sites, and degradation characteristics that contribute to stable long‐term culture conditions. The performance differences observed between fibrin gel and other commercially available matrices, including Matrigel and collagen‐based systems, underscore the importance of careful matrix selection in tissue engineering approaches. Notably, hydrogel evaluation in this study was primarily based on mesothelial cell distribution, assessed via light microscopy, and cell viability, evaluated through live/dead staining. While these observations provided a robust qualitative assessment and guided the choice of fibrin hydrogels, the analysis remains largely descriptive. Quantitative comparison of additional hydrogel characteristics could further strengthen matrix selection. Future studies should include systematic, quantitative assessments to validate and refine hydrogel optimization. Osório et al. in 2021 described a variety of commercially available hydrogels where each hydrogel composition had unique advantages and disadvantages.^16^ Especially, the formation of a mesothelial monolayer was identified as a crucial event for peritoneal membrane physiology.^2^ Collagen‐based hydrogels (RatCol and FibriCol) exhibited dimensional contraction during culture, which affected their mechanical stability (Figure 3). This shrinkage phenomenon has been reported in other tissue engineering studies and remains a technical challenge for collagen‐based matrices.^17^

The observed improvement in cell viability under perfused conditions supports the value of incorporating fluid dynamics into in vitro models. The peritoneum naturally experiences continuous fluid turnover, and our results suggest that static conditions may not recapitulate this aspect of peritoneal biology. Beyond enhanced cell survival, perfusion allows real‐time sampling of secreted factors, metabolites, and therapeutic compounds, potentially enabling pharmacokinetic studies and biomarker discovery applications that are challenging to achieve with static systems.

Platform characterization through LDH analysis, immunofluorescence, and histological staining validated the microfluidic platform's functionality. The LDH assay demonstrated here provides a foundation for evaluating pharmaceutical interventions on peritoneal tissue through established cell viability methodologies.^18^, ^19^ H&E staining revealed comparable tissue architecture between the microfluidic platform and native peritoneal tissue (Figure 3), successfully recapitulating key peritoneal features. However, several limitations warrant consideration. The use of immortalized MeT5A cells, though common in mesothelial research, may introduce cellular alterations affecting differentiation capacity and DNA damage responses compared to primary cells.^20^, ^21^ Future iterations incorporating primary human mesothelial cells could enhance physiological relevance and enable patient‐specific modeling approaches for personalized medicine applications, potentially providing deeper insights into individual pathophysiology. Moreover, while the fibroblasts used in our platform were patient‐derived, only a single line was employed. Expanding the study to include additional patient‐derived fibroblast lines will be important to evaluate inter‐patient variability and to strengthen the generalizability of our findings. The present study focuses on the proof‐of‐concept validation of a PDMS‐free microfluidic peritoneal adhesion model. The established platform now provides the opportunity to investigate the mechanisms of adhesiogenesis in greater detail, including analyses of adhesion‐associated markers, cytokine profiles, and transcriptomic changes in future studies. In addition, as the platform enables co‐culture of mesothelial and stromal cells, it may enable the investigation of dynamic crosstalk mechanisms during adhesion development. The absence of immune components represents another area for enhancement. Given the peritoneum's crucial role in immune surveillance and the importance of inflammation in both adhesion formation and metastatic seeding, incorporating immune cells could significantly expand the platform's modeling capabilities.^2^ Future iterations might also benefit from incorporating vascular components to model the peritoneal microcirculation more completely. The integration of endothelial cells and perfusable vasculature would enable more sophisticated studies of barrier function and drug transport. Despite these limitations, our platform offers significant translational opportunities for peritoneal disease research. The adhesion model addresses a critical clinical need, given that adhesions develop in 67%–93% of patients following abdominal surgery and current preventive strategies remain inadequate.^10^, ^22^ This 3D system may enable systematic evaluation of anti‐adhesion therapeutics under controlled conditions. The platform's versatility extends beyond adhesion modeling to other peritoneal pathologies, including carcinomatosis, metastasis, endometriosis and inflammation. These applications have the potential to enhance understanding of disease mechanisms and to accelerate therapeutic development through physiologically relevant in vitro modeling. In the future, the developed microfluidic platform could be further refined to support fully individualized systems, thereby providing a translational framework for personalized medicine approaches.

## 4 METHODOLOGY

Permission to use the vector in Figure 5a and the petri dish in Figure 5d was obtained from BioRender.com.

### 4.1 Cell culture

Patient‐derived peritoneum for the isolation of primary peritoneal fibroblasts (hpFib) was obtained from consenting patients undergoing a cesarean (Ethical approval: 495‐2018BO2, approval: 25.09.2018). Written informed consent was obtained from all patients. Primary fibroblasts and the mesothelial cell line MeT5A (*Homo sapiens*, male, mesothelium, RRID: CVCL_3749) were cultured under sterile conditions and maintained in a humidified incubator at 37°C and 5% CO_2_. MeT5A cells were obtained from ATCC (#CRL‐9444) in 2022. The cell line was authenticated by the supplier using short tandem repeat (STR) profiling and has not been reported as misidentified or contaminated. ATCC confirms that all distributed cell lines are free of mycoplasma contamination at the time of shipment. No additional authentication or mycoplasma testing was performed in this study. For maintenance, cells were first washed twice with DPBS (Dulbecco's Phosphate Buffered Saline, PAN‐Biotech, #P04‐36500). Afterwards, detachment was performed using an adequate amount of trypsin–EDTA (0.05%; Gibco™, #25300062) and the cell solution was then resuspended in cell culture medium (DMEM for hpFib, Dulbecco's Modified Eagle Medium, Gibco™, #41965062 or Medium 199 for MeT5A, Earle's Salts, Gibco™ #11150059). Both cell culture media were supplemented with 10% FCS (Fetal bovine serum, Gibco™, #A5256701) and 1% Penicillin/Streptomycin (10.000 U/mL; Gibco™, #15140122). M199 additionally contained 1% insulin‐transferrin‐selenium (ITS‐G, 100X, Gibco™, #41400045), 0.1% trace elements B (1.000X, Corning™, #25‐022‐CI), 400 nM hydrocortisone (Sigma‐Aldrich Merck, #H0888‐1G), and 10 ng/mL hEGF (Gibco™, #PHG0313). A desired volume of the cell suspension was retained in the cell culture flask. For 2D experiments, 15 625 hpFib/cm^2^ and 31 250 MeT5A/cm^2^ were seeded in well plates.

### 4.2 Fabrication of microfluidic platform

The microfluidic platform was assembled according to Kromidas et al.^19^ All parts used for the fabrication were supplied by the μOrganoLab of the Natural and Medical Sciences Institute (NMI) in Reutlingen, Germany.

Polyethylene terephthalate (PET) membranes underwent a cleaning process by submersion in isopropanol (MicroChemicals, #MIPU1025) followed by a drying process and were subsequently aligned over the media channel of the styrene‐ethylene‐butylene‐styrene/polycarbonate (SEBS/PC) media layer. The SEBS tissue layer was aligned on top of the assembly and thermally bonded in an oven (Memmert) at 60°C for 12 h. Polymethyl methacrylate (PMMA) media reservoir layers were aligned onto the stack of polymer layers followed by another bonding step at 60°C for at least 12 h.

For perfusion, cannulas from stain‐less steel plastic hub dispensing needles (Gauge 23, Weller, #KDS2312P) were cut to a length of approximately 5 mm (outer diameter of 0.6 mm) and inserted into flexible plastic tubing (VWR, #AAD04103) with a length of approximately 8 mm (inner diameter of 0.5 mm). The plastic tubing of these adapters was inserted into the in‐ and outlet ports of the microfluidic platform. To seal the connection, polydimethylsiloxane (PDMS) was applied and cured for 1–2 h at 60°C. Prior to the experiments, the platform was subjected to a sterilization process with 70% EtOH followed by washing with DI water and equilibration at 37°C.

### 4.3 Hydrogels and 3D cell seeding

Several hydrogels were tested concerning the distribution and viability of the MeT5A cell layer. All hydrogels were seeded containing hpFib with a final concentration of 1 × 10^6^ cells/mL.

#### 4.3.1 Dextran hydrogel

Three dimensional Life Dextran‐CD Hydrogel SG (Cellendes GmbH, #G93‐1) and 3‐D Life RGD Peptide (1 μmol) (Cellendes GmbH, #09‐P‐001) were chemically crosslinked according to the manufacturer's instructions.

#### 4.3.2 Fibrin gel

Bovine Fibrinogen (Sigma Aldrich, #341573) was reconstituted in DPBS at a concentration of 6 mg/mL and stored at −80°C. For experiments, diluted fibrinogen was equilibrated at 37°C. Cell suspension was mixed with human thrombin (Sigma‐Aldrich Merck, #605190) with a final thrombin concentration of 3 U/mL. Fibrinogen was added at a ratio of 1:1. The seeded fibrin gel was incubated for approximately 5 min at 37°C and 5% CO_2_.

#### 4.3.3 Matrigel, FibriCol®, RatCol®, HyStem®‐C

Matrigel (Cultrex™ Reduced Growth Factor Basement Membrane Extract Type 2, Pathclear, rnd. systems, #3533‐005‐02), FibriCol® (Advanced BioMatrix, #5133), RatCol® (Advanced BioMatrix, #5153) and HyStem®‐C (Advanced BioMatrix, #GS313) were prepared according to the manufacturer's instructions and incubated for 20 min at 37°C and 5% CO_2_. Incubation time for Matrigel was 30 min.

The following day, MeT5A were seeded twice onto hydrogel, with the concentration for each seeding ranging from 0.15 × 10^6^ cells/cm^2^ to 1 × 10^6^ cells/cm^2^, as specified for each experiment.

### 4.4 Tissue retrieval

For analysis, tissue retrieval was performed using a 4 mm biopsy punch (pfm medical AG, #49401). Tissue surrounded by a ring of SEBS material was removed from the biopsy punch with tweezers and transferred to 96‐well plates.

### 4.5 Live/dead staining

Samples were washed with DPBS+. After tissue retrieval, samples were stained in a 96‐well plate. Calcein (Cayman Chemical, #Cay14948‐1) and Hoechst33342 (Thermo Scientific™, #62249) were incubated at a concentration of 1:500 in DPBS+ for 20 min at 37°C. Cells were washed again and incubated for 5 min at 37°C and 5% CO_2_ with Propidium Iodide (PI) (Sigma‐Aldrich, #P4170) diluted 1:20 in DPBS+. Cells were washed with DPBS+ before fixing with ROTI®Histofix.

### 4.6 Immunofluorescence staining

#### 4.6.1 Mesothelial cell and fibroblast markers

Samples were washed with DPBS+ (Dulbecco's Phosphate Buffered Saline, PAN‐Biotech, #P04‐35500) before fixation with ROTI®Histofix (Carl Roth, #P087.6) for 30 min. Cells were permeabilized with 3% BSA (Sigma Aldrich, #A9647) and 0.1% Triton™X‐100 (Sigma‐Aldrich, #9036‐19‐5) diluted in DPBS for 30 min. Prior to staining, cells underwent another washing process with DPBS. Primary antibodies were diluted as described in Tables S1 and S2 in 3% BSA in DPBS and added to the samples and incubated overnight at 4°C. After washing with DPBS, secondary antibodies were diluted as described in Table S3 together with Hoechst33342, diluted 1:500, in 3% BSA in DPBS and incubated at room temperature for 45 min. Fluorescence was observed under the fluorescence microscope (Axio Observer 7, Zeiss).

#### 4.6.2 ZO‐1

Samples were washed with DPBS+ followed by fixation with 100% methanol (Merck, #106009) at −20°C for 10 min. Subsequently, samples were washed with DPBS. Blocking was performed with 10% FCS and 1% BSA in DPBS for 30 min. ZO‐1 antibody (ThermoFisher, #40‐2200) was diluted 1:200 in the blocking solution and incubated overnight at 4°C. Samples were washed with 0.1% Tween 20 (Sigma‐Aldrich Merck, #P1379‐1L) in DPBS. Alexa Fluor 647™ (ThermoFisher, #A‐31573) was diluted 1:200 in the blocking solution with Hoechst33342 diluted 1:500 for an incubation time of 45 min at room temperature. Washing with 0.1% Tween 20 in DPBS was repeated. Samples were observed using a fluorescence microscope (Axio Observer 7, Zeiss).

### 4.7 Cell viability assays

An LDH assay (LDH‐Glo™ Cytotoxicity Assay, Promega, #J2381) was conducted following the manufacturer's instructions to assess cell viability based on LDH release by plasma membrane damage. Effluent of 3D samples was diluted 1:10 in LDH storage buffer and stored at −20°C. Luminescence was quantified after an incubation time of 60 min via a plate reader (1 s per well, Varioskan™ LUX multimode microplate reader, Thermo Scientific™, #VL000D0).

The colorimetric cell proliferation assay CellTiter 96® AQueous One Solution Cell Proliferation Assay (MTS assay, Promega, USA, #G3581) was performed according to the manufacturer's instructions. Samples were incubated for 2 h at 37°C and 5% CO_2_. Readout was performed via a plate reader (Varioskan™ LUX multimode microplate reader, Thermo Scientific™, #VL000D0).

### 4.8 HE staining

Samples were fixed with Bouin's fixative (Carl Roth, #6482.3). PC layer was removed from the platform with tweezers and tissue retrieval was performed, before the samples were embedded in HistoGel™ (Thermo Scientific, #HG‐4000‐012). Samples were then embedded in paraffin and cut into approximately 7‐μm‐thick sections. Following dewaxing and rehydration, hematoxylin (Sigma Aldrich, #51275)‐eosin (Carl Roth, #3137.2) (HE) staining was carried out. Images were obtained using a microscope (EVOS™ M7000 Imaging System, Invitrogen™, #AMF7000).

### 4.9 Image acquisition and analysis

Images were acquired using an inverted fluorescent microscope (Axio Observer 7, Zeiss), if not stated otherwise. Processing of images, including the adjustment of brightness and contrast, were performed using ZEN lite Software (Version 3.4, Zeiss) and ImageJ 1.52a (National Institutes of Health and the Laboratory for Optical and Computational Instrumentation [LOCI, University of Wisconsin]), while applying the same settings across all images within an experiment.

### 4.10 Establishment of a stable MeT5A_luc cell line

Stable transfection of MeT5A cells with the luciferase‐containing plasmid pNLCol3[luc2‐P2A‐NlucP/CMV/Hygro] (Promega, #TM426) was performed chemically with polyethylenenimine (PEI; PEI STAR™ transfection reagent, R&D Systems, #7854/100). For transfection, 5 × 10^4^ cells/cm^2^ were seeded into a T25 cell culture flask and cultured overnight. The plasmid DNA and PEI were mixed 1:3, briefly vortexed and incubated for 20 min at room temperature. The DNA:PEI complexes were added dropwise to the cells and incubated for 48 h at 37°C and 5% CO_2_. Medium without supplements was then changed to the selection medium (consisting of DMEM with 10% FCS, 1% P/S and 70 μg/mL hygromycin [ThermoFisher Gibco™, #10687010]). After 2 weeks of selection, the transfected MeT5A_luc cells were expanded and used for experiments.

### 4.11 Bead‐adhesion‐assay

Dextran beads (Cytiva, # GE17‐0485‐01) were prepared as described by the manufacturer. 1.04 × 10^5^ MeT5A_luc cells/cm^2^ were mixed with 140 μL/mL dextran beads and cultured for 96 h at 37°C and 5% CO_2_ in a 96‐well plate coated with hydroxyethylmethacrylate (HEMA, Sigma‐Aldrich, #P3932). After mesothelial cell seeding on the 3D microfluidic platform, medium was carefully removed from the wells of the 96‐well plate and the MeT5A_luc cells cultured on the dextran beads were treated with talcum solved in DMEM (1 mg/mL, VWR Chemicals, #83557.260) as an adhesion inducer. Beads were then transferred to the untransfected MeT5A cells seeded on the microfluidic platform and were incubated at 37°C and 5% CO_2_ for 6 and 24 h.

### 4.12 Luciferase‐assay

The Nano‐Glo® Dual‐Luciferase® Reporter Assay System (Promega, #N1610) was used to measure luciferase activity and was performed according to the manufacturer's instructions. ***

In brief, the cell culture medium was removed, and each well was washed three times with DPBS. An adequate amount of medium was added per well and mixed 1:1 with ONE Glo™ EX Reagent and was shook for 3 min at 300 rpm. Then firefly luciferase luminescence measurement was performed with an integration time of 1 s on the plate reader.

### 4.13 Data analysis

For data presentation and statistics GraphPad Prism 9.3.1 (GraphPadSoftware Inc.), Microsoft PowerPoint (Microsoft Office LTSC Professional Plus 2024, Version 2408, Microsoft Corporation) and BioRender (Science Suite Inc.) were utilized. Data are shown as mean ± standard deviation (SD) and individual values for each replicate are indicated with sample sizes (*n*) specified in each figure legend. Data were analyzed using GraphPad Prism 9.3.1. For testing statistical significance, student's *t* test or one‐way ANOVA were utilized. *p*‐values smaller than 0.05 were referred to as statistically significant (*).

## 5 CONCLUSION

We have successfully engineered the first comprehensive microfluidic model of human peritoneum that bridges the gap between oversimplified cell cultures and complex animal models. This platform not only advances our fundamental understanding of peritoneal biology but also provides a powerful tool for drug discovery and therapeutic development.

## AUTHOR CONTRIBUTIONS

Katharina Peisert and Franziska Keßler contributed equally to this work. Martin Weiss and Peter Loskill conceived the study. Adrian Weghofer and Hui‐Yu Liu designed and fabricated chips. Katharina Peisert and Franziska Keßler performed the in‐vitro experiments and the analysis of the experimental results. Jan Pauluschke‐Fröhlich, Sara Y. Brucker, Peter Jakubowski, Felix Neis, Bernhard Krämer and Jürgen Andress provided the tissue samples. Katharina Peisert, Franziska Keßler and Martin Weiss wrote the manuscript. Martin Weiss supervised the study and secured funding.

## CONFLICT OF INTEREST STATEMENT

All other authors declare that they have no competing interests.

## Supporting information


**Data S1:** Supporting information.

## Data Availability

The data that support the findings of this study are available from the corresponding author upon reasonable request.
