# Supplementary material for: Engineering human peritoneum in vitro: A novel microfluidic platform for modeling peritoneal physiology and pathophysiology
Source: Bioeng Transl Med. 2026 Mar 9;11(4):e70128. doi: 10.1002/btm2.70128 (PMC13327602; doi:10.1002/btm2.70128)
Supplement: Supplementary file 1 — Data S1: Supporting information. [file BTM2-11-e70128-s001.docx]

Engineering Human Peritoneum in Vitro: A Novel Microfluidic Platform for Modeling Peritoneal Physiology and Pathophysiology

Katharina Peisert¹‡, Franziska Keßler¹‡, Sara Y. Brucker¹, Jan Pauluschke-Fröhlich¹, Peter Jakubowski^1^, Felix Neis¹, Bernhard Krämer¹, Jürgen Andress¹, Adrian Weghofer*^2^*, Hui-Yu Liu^2^, Peter Loskill^2,3^, Martin Weiss^1,3^*

**Supporting table S1: Overview over utilized primary antibodies.**

| **Antibody** | **Species** | **Manufacturer** | **Catalog number** | **Dilution used for 2D experiments** | **Dilution used for 3D experiments** |
| --- | --- | --- | --- | --- | --- |
| Anti-Calretinin | Goat | rnd systems, USA | AF5065 | 1:50 | 1:50 |
| Anti-Cytokeratin Broad Spectrum (AE1 & AE3) | Mouse | Zytomed Systems, Germany | MSK019 | 1:50 | 1:50 |
| Anti-Fibronectin | Rabbit | Abcam, UK | Ab2413 | 1:200 | 1:200 |
| Anti-Mesothelin | Rabbit | Invitrogen, USA | PA5-79697 | 1:250 | N/A |
| S100A4 Polyclonal Antibody (Anti-FSP-1) | Rabbit | Invitrogen, USA | PA5-95736 | 1:200 | N/A |
| Wilms‘ tumor protein (WT1) antibody | Rabbit | SantaCruz, USA | Sc-192 | 1:100 | 1:50 |
| ZO-1 antibody | Rabbit | ThermoFisher, USA | 40-2200 | 1:200 | 1:200 |

**Supporting table S2: Overview over utilized primary conjugated antibodies.**

| **Antibody** | **Species** | **Manufacturer** | **Catalog number** | **Excitation [nm]** | **Emission [nm]** | **Dilution used for 2D experiments** | **Dilution used for 3D experiments** |
| --- | --- | --- | --- | --- | --- | --- | --- |
| Anti-human CD90 (Thy1), conjugated with APC/Cyanine7 | Mouse | BioLegend, USA | 328132 | 633 | 775-785 | 1:50 | 1:50 |

**Supporting table S3: Overview over utilized secondary antibodies.**

| **Antibody** | **Species** | **Manufacturer** | **Catalog number** | **Excitation [nm]** | **Emission [nm]** | **Dilution used for 2D experiments** | **Dilution used for 3D experiments** |
| --- | --- | --- | --- | --- | --- | --- | --- |
| Anti-Goat IgG (H+L) Cross-Adsorbed Secondary Antibody Alexa Fluor^TM^ 546 | Donkey | ThermoFisher, USA | A-11056 | 561 | 572 | 1:500 | N/A |
| Anti-Mouse IgG (H+L) Cross-Adsorbed Secondary Antibody Alexa Fluor^TM^ 647 | Goat | ThermoFisher, USA | A-21235 | 650 | 671 | 1:200 | 1:200 |
| Anti-Rabbit IgG H&L (Alexa Fluor® 488) | Goat | Abcam, UK | Ab150077 | 495 | 519 | 1:500 | 1:250 |
| Anti-Rabbit IgG (H+L) Highly Cross-Adsorbed Secondary Antibody, Alexa Fluor™ 647 | Donkey | ThermoFisher, USA | A-31573 | 650 | 671 | 1:200 | 1:200 |
| Anti-Rabbit IgG H&L, Alexa Fluor® 555 | Goat | Abcam, UK | Ab150078 | 555 | 565 | 1:200 | 1:200 |


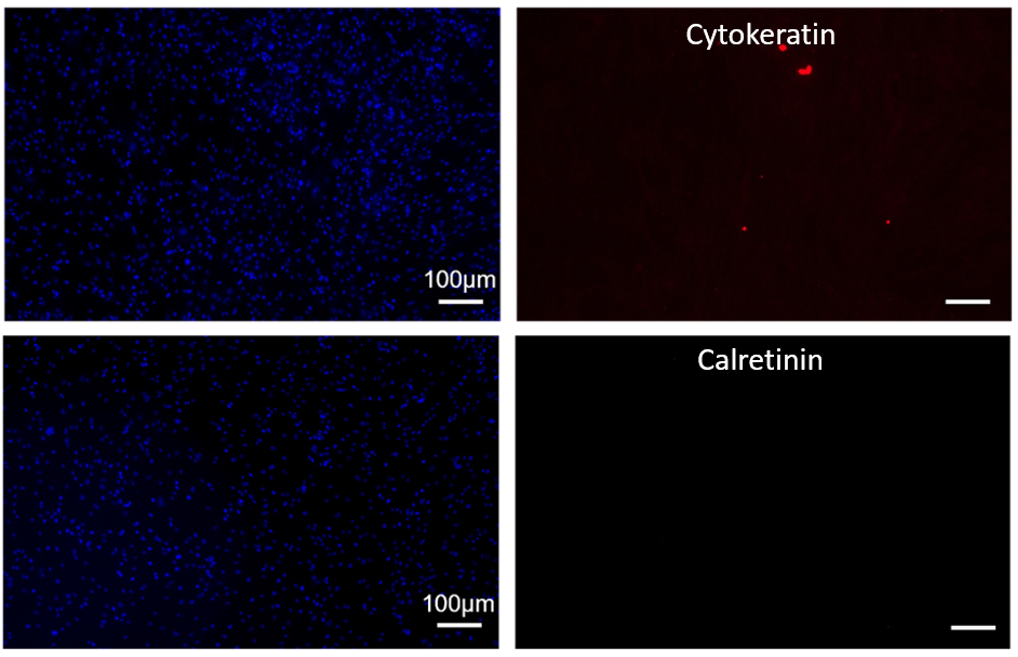

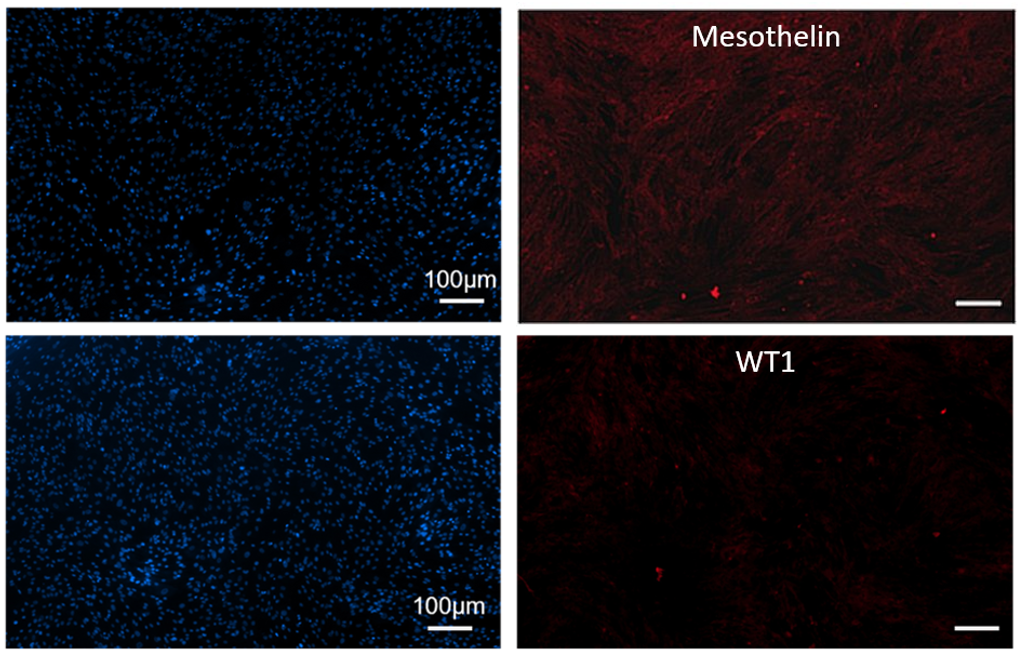


**Supporting figure S1: Antibody Staining of mesothelial cell markers cytokeratin, calretinin, mesothelin and WT1 in hpFib in 2D.** hpFib were cultured for 24 h. Cytokeratin and calretinin were not expressed in hpFib. The expression level of WT1 was very low. Mesothelin showed an unspecific expression in hpFib. Scale bar: 100 µm.


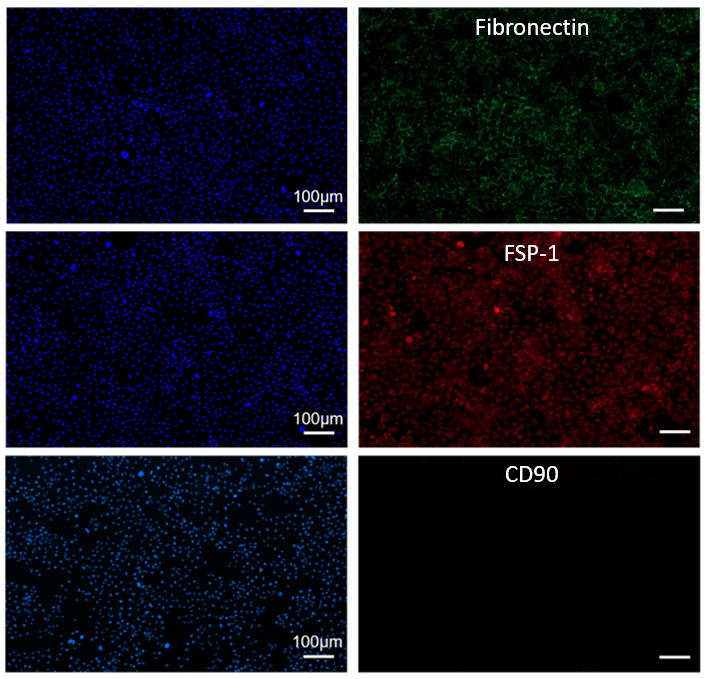


**Supporting figure S2: Antibody Staining of fibroblast markers fibronectin, FSP-1 and CD90 in MeT5A in 2D.** MeT5A were cultured for 24 h. Fibronectin shows a weaker signal than in hpFib. FSP-1 exhibits a strong cytoplasmic signal in MeT5A. CD90 was not expressed in MeT5A. Scale bar: 100 µm.

**Supporting figure S3: Quantification of calretinin, FSP-1, WT-1, cytokeratin, fibronectin, mesothelin and CD90 in MeT5A and hpFib in 2D.** The fluorescence signals of the antibody staining were quantified in ImageJ. Three representative images were analyzed for each condition. The cells were identified as regions of interest (ROIs) based on the Hoechst signal, and the total fluorescence within these ROIs was measured using the “Analyze Particles” function. The measured antibody signals were normalized to the respective Hoechst signal and expressed as a percentage to represent the relative signal intensity per image. Data are presented as mean ± SD, and individual values for each replicate are indicated (n=3), *p < 0.05 (as determined by t-test).
